# Anatomical Considerations for Thread Lifting Procedure

**DOI:** 10.1111/jocd.16618

**Published:** 2024-10-08

**Authors:** Gi‐Woong Hong, Soo‐Bin Kim, Youngjin Park, Soo Yeon Park, Lisa Kwin Wah Chan, Kar Wai Alvin Lee, Olena Sydorchuk, Jovian Wan, Kyu‐Ho Yi

**Affiliations:** ^1^ Sam Skin Plastic Surgery Clinic Seoul Korea; ^2^ Division in Anatomy and Developmental Biology, Department of Oral Biology Human Identification Research Institute, BK21 FOUR Project, Yonsei University College of Dentistry Seoul Korea; ^3^ Obliv Clinic Incheon Korea; ^4^ Made‐Young Plastic Surgery Clinic Seoul Korea; ^5^ EverKeen Medical Centre Hong Kong China; ^6^ Wlabel Seoul Korea; ^7^ Sihler Inc. Seoul South Korea; ^8^ Maylin Clinic (Apgujeong) Seoul Korea

**Keywords:** aesthetic procedures, complications, facial anatomy, surgical techniques, thread lifting

## Abstract

**Background:**

Thread lifting is a minimally invasive procedure that enhances facial aesthetics by repositioning sagging tissues with absorbable threads. It requires a comprehensive understanding of facial anatomy for safe and effective results.

**Aims:**

This study aims to highlight the critical anatomical considerations in thread lifting, including the navigation of facial vascular structures, the protection of facial nerves, manipulation of fat compartments, and engagement of retaining ligaments. These factors are essential for minimizing complications and achieving optimal outcomes.

**Patients/Methods:**

A review was conducted focusing on the anatomical elements critical to thread lifting. The study analyzed clinical outcomes related to vascular structures, nerve pathways, fat compartments, and ligaments in patients undergoing the procedure.

**Results:**

The review revealed that careful navigation of facial blood vessels is crucial to avoid complications such as bleeding and bruising. Knowledge of facial nerve pathways is essential to prevent nerve damage, which could result in facial weakness or paralysis. Proper manipulation of facial fat compartments helps address aging‐related changes, and engaging retaining ligaments is vital for a sustainable lift without tissue distortion.

**Conclusions:**

Thread lifting demands not only technical skill but also a deep understanding of facial anatomy to ensure patient safety and desired aesthetic results. Expertise in these anatomical considerations is essential for minimizing complications and preserving the natural function of facial structures.

## 1 Introduction

Thread lifting is an increasingly popular non‐surgical procedure aimed at enhancing facial aesthetics through the strategic placement of absorbable threads. These threads, which can be smooth, twisted, or barbed, are introduced into the skin and deeper layers to reposition and tighten the tissue. As the procedure gains popularity for its minimally invasive nature and potential for dramatic results, understanding the fundamental anatomical considerations is crucial for safe and effective practice.

This review article delves into the essential anatomical structures involved in thread lifting, with a particular focus on vessels, nerves, fat compartments, and retaining ligaments. The complexity of facial anatomy demands a meticulous approach to avoid vital structures such as vessels and nerves, which can significantly impact the outcomes of the procedure. Special attention is given to the vascular anatomy, including the risks associated with navigating near major arteries and veins which, if accidentally compromised, could lead to severe complications such as hematoma or more severe bleeding episodes.

Moreover, the intricate interaction between facial nerves and the thread lifting technique is discussed, highlighting strategies to minimize nerve trauma, which can result in temporary or permanent facial weakness. The article also explores the role of facial retaining ligaments and fat compartments in achieving desired aesthetic results, emphasizing the importance of understanding their behavior during aging and their response to lifting techniques.

Through a comprehensive review of current literature and clinical practices, this article aims to equip practitioners with the knowledge necessary to perform thread lifts safely and effectively, ensuring patient satisfaction and minimizing the risk of complications.

## 2 Supra‐SMAS Thread Placement

In facial thread lifting, the recommended placement of threads is typically within the supra‐SMAS (Superficial Musculoaponeurotic System) layer, which is located above the SMAS and below the subcutaneous fat. This layer is preferred because it helps avoid major nerves and arteries, such as the facial nerve branches and the transverse facial artery, thereby reducing the risk of complications. The supra‐SMAS placement allows for effective lifting and contouring without significant risk to critical structures [1].

However, the placement of threads can also be altered into the subSMAS or intraSMAS layers, depending on the specific aesthetic goals and anatomical considerations. The subSMAS layer, which lies just beneath the SMAS, can provide a deeper lift, suitable for patients with more pronounced sagging or thicker skin. This deeper approach requires careful consideration of the underlying structures to avoid potential complications.

Similarly, intraSMAS placement, which involves inserting threads within the SMAS layer itself, offers another variation that can be tailored to individual patient needs. This technique allows for a more integrated lift, working directly with the SMAS to enhance support and firmness. The choice between supraSMAS, subSMAS, and intraSMAS placements depends on factors such as the patient's skin thickness, the degree of sagging, and the desired outcome [2, 3, 4, 5].

**FIGURE 1:**
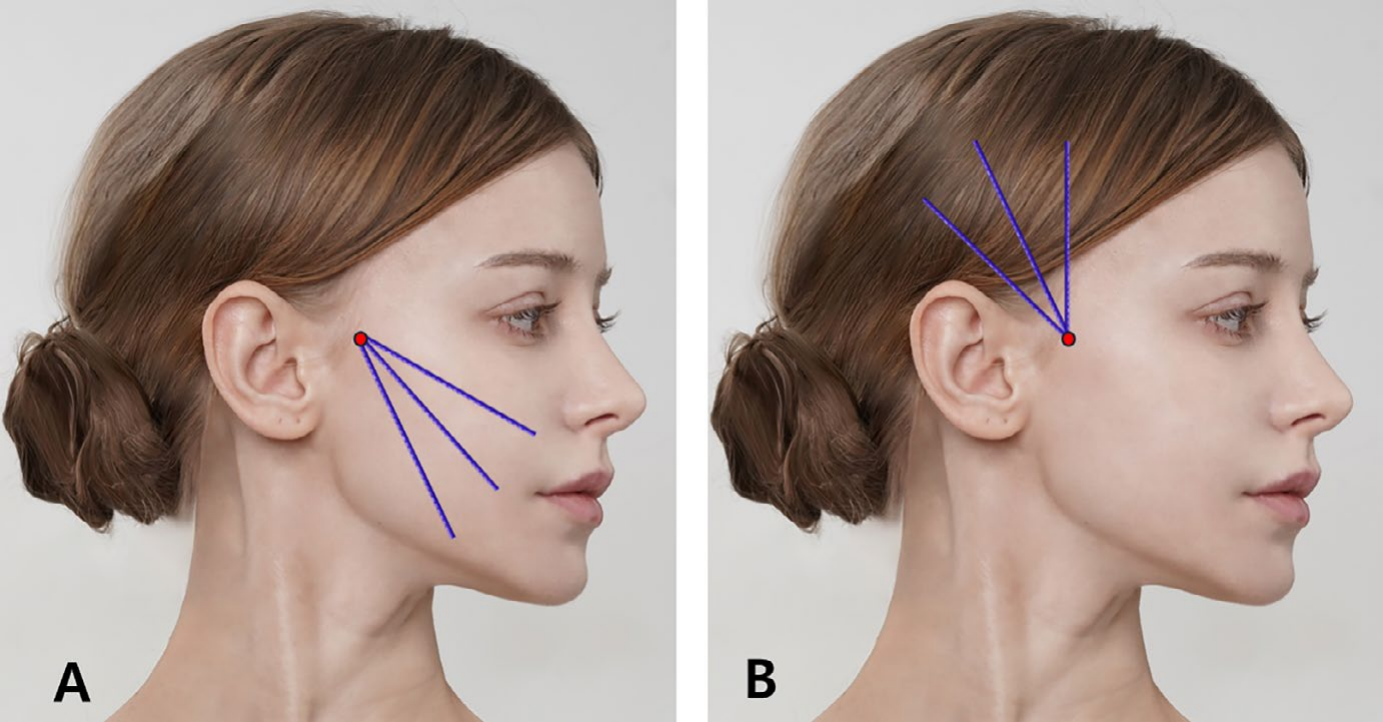
Design of short‐ or medium‐length I‐type bidirectionalcogged threads. Panel (A) represents antegrade approach of the threads while (B) represents retrograde approach of thread lifting. The thread depicted in the image is the Secret illusion 10 cm long bidrectional thread and LVDR (Hyundaemeditech Inc., Wonju, Republic of Korea and Sihler Inc., Seoul, Republic of Korea) which has benefits in retrograde approaches.

The antegrade approach, involving the insertion of threads from a distal point (such as the temporal region or hairline) to a proximal anchor point (such as the midface or jawline), creates an upward and backward lift. The retrograde approach, starting from areas requiring lifting (like the nasolabial fold or marionette lines) and pulling back toward an anchoring point, allows for precise control over thread tension and positioning. Both approaches focus on the supraSMAS layer but can be adapted for subSMAS or intraSMAS placements as needed (Figure 1).

Floating type threads, used primarily for subtle lifting and contouring, are typically placed in the supraSMAS layer but can also be adapted to subSMAS or intraSMAS placements for a more comprehensive approach. These threads enhance skin firmness and elasticity and are particularly useful in combination with other thread types. The bidirectional thread (Hyundaemeditech Inc., Seoul, Republic of Korea) effectively pulls subcutaneous tissue upward and laterally.

### 2.1 Vessels

Thread lifting, similar to filler injections, requires careful handling of vascular structures to prevent complications. While thread lifting does not typically cause vascular complications like fillers, significant bleeding can occur if major veins or arteries are disturbed during the procedure. This can complicate the treatment and lead to severe swelling and bruising.

#### 2.1.1 Temporal Region

Specifically, while the lower face generally has superficially located vessels that pose less risk if handled carefully, areas like the temples or upper face where threads are anchored have a higher likelihood of damaging important vessels. These vessels often run alongside sensory nerves, so if a patient reports sharp, severe pain, it is safer to retract the needle or cannula slightly and adjust the insertion angle and depth.

**FIGURE 2:**
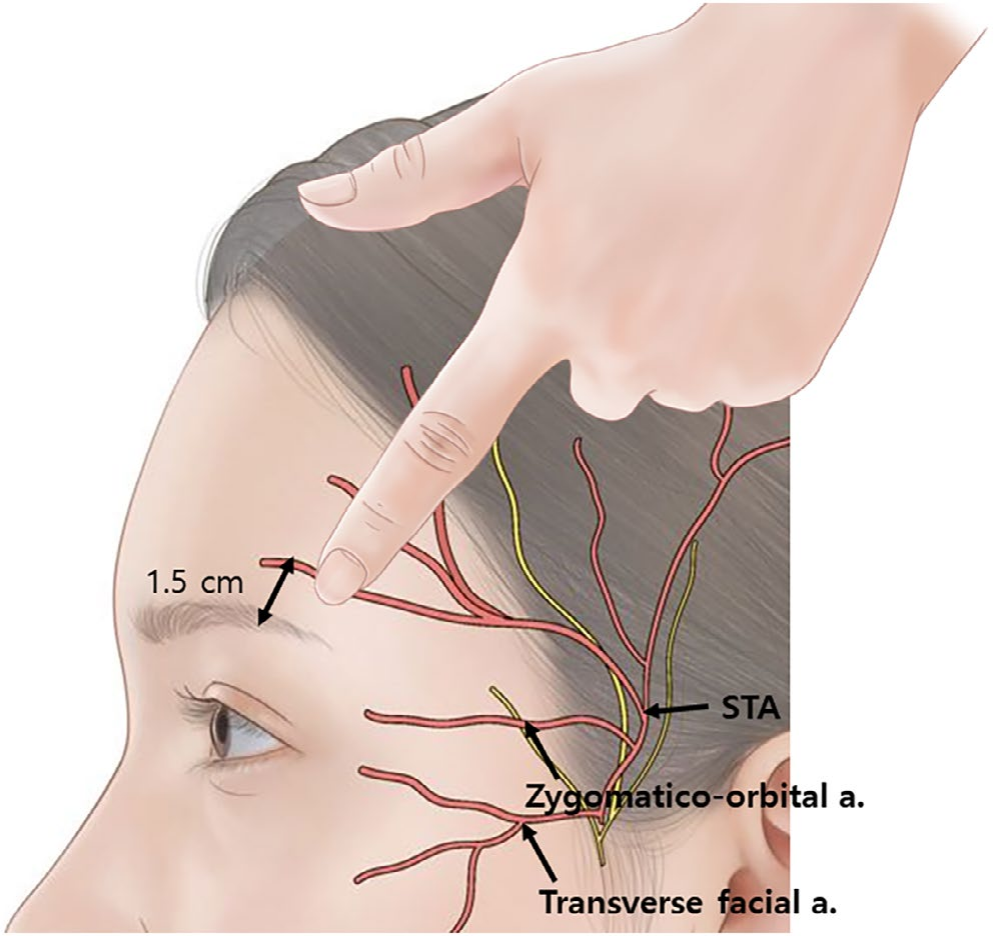
The shift in depth typically occurs near the outer boundary of the eyebrow and the lateral canthal line, where smaller branches of the STA may be present subcutaneously. Thus, understanding and cautious handling of these vascular paths are essential to minimize the risk of significant complications during thread lifting procedures.

Thin PDO monofilament threads may cause bleeding and bruising as they pierce through skin multiple times, primarily affecting capillaries and small veins in the subcutaneous fat layer; however, these bleedings are usually minor and can be quickly managed by applying pressure. In contrast, using thicker barbed threads requires careful placement of entry points near the hairline in front of the ears or the temples, where large vessels are commonly located, especially as the threads pass through or deeper than the SMAS layer. A critical vessel in these areas is the superficial temporal artery (STA), which ascends in front of the ear following a crease line, bifurcating at about the level of the superior orbital rim—more often above it than below. After bifurcation, the anterior branch of the STA travels at an average angle of 60.8° toward the superomedial direction along the outer boundary of the frontalis muscle. As it progresses toward the forehead, it becomes more superficial. The shift in depth typically occurs near the outer boundary of the eyebrow and the lateral canthal line, where smaller branches of the STA may be present subcutaneously. Thus, understanding and cautious handling of these vascular paths are essential to minimize the risk of significant complications during thread lifting procedures (Figure 2) [6].

Even if the approximate anatomical location of the STA is known, there is still variability in its path, which can occasionally lead to the artery being punctured during lifting procedures using fixed barbed threads. Typically, a thick temporal needle is used to anchor these threads in the temple area. If slight bleeding occurs, it's likely that a vein or small vessel was punctured, but if the STA is pierced, the temple area may swell and blood may pulse out through the needle hole within 1–2 s. In such cases, it is crucial to apply pressure for about 3–5 min to ensure proper hemostasis before choosing a new entry point in a different area to continue the procedure. If hemostasis is not achieved with pressure alone, a thick nylon suture may be used to temporarily stitch the surrounding skin and tissue around the vessel until the bleeding completely stops [7].

In the area of the temples and the hairline in front of the ears, the superficial temporal vein (STV) is also present. Unlike an artery, the STV does not cause pulsatile bleeding, but if damaged, it can lead to significant bruising. Therefore, it is crucial to handle this area gently during procedures to avoid damaging the vein and minimize the risk of bruising [8].

#### 2.1.2 Transverse Facial Artery

The transverse facial artery (TFA) is a significant arterial branch that primarily supplies blood to the lateral face, including the parotid gland, parotid duct, facial nerve, facial muscles, and overlying skin. As described by Yang et al. [9], the TFA commonly originates from the superficial temporal artery within the parotid gland or at its upper border. It typically branches into superior and inferior trunks, which further divide to supply various facial structures. The superior trunk often supplies the malar area and masseter muscle, crossing above the parotid duct, while the inferior trunk may supply the lower parotid region and adjacent muscles. The TFA is crucial for procedures involving facial aesthetics and reconstruction due to its role in vascularizing both superficial and deep structures of the lateral face. Additionally, the artery's anatomical variations, such as the number of branches and the presence of perforators, are important considerations for surgical planning and the prevention of iatrogenic injuries.

#### 2.1.3 Zygomatico‐Orbital Artery

The zygomatico‐orbital artery (ZOA) is a significant vascular structure primarily involved in supplying blood to the lateral canthal region and the suprazygomatic territory. According to Park et al. [10], the ZOA typically bifurcates from the frontal branch of the superficial temporal artery (STA) and may occasionally originate from other branches such as the middle temporal or parietal branches of the STA. Present in approximately 85% of cases, the ZOA is crucial for various cosmetic and reconstructive procedures involving the temporal and periorbital regions. The artery runs in close proximity to the line connecting the tragus and the superciliary arch (TR‐SA line), often within a narrow margin, which necessitates careful consideration during procedures like filler injections or flap surgeries to avoid vascular complications. The anatomical course of the ZOA, including its relationship with the STA and other nearby structures, is vital for clinicians to understand, ensuring safer and more effective surgical interventions in the temporal area.

#### 2.1.4 Facial Artery

**FIGURE 3:**
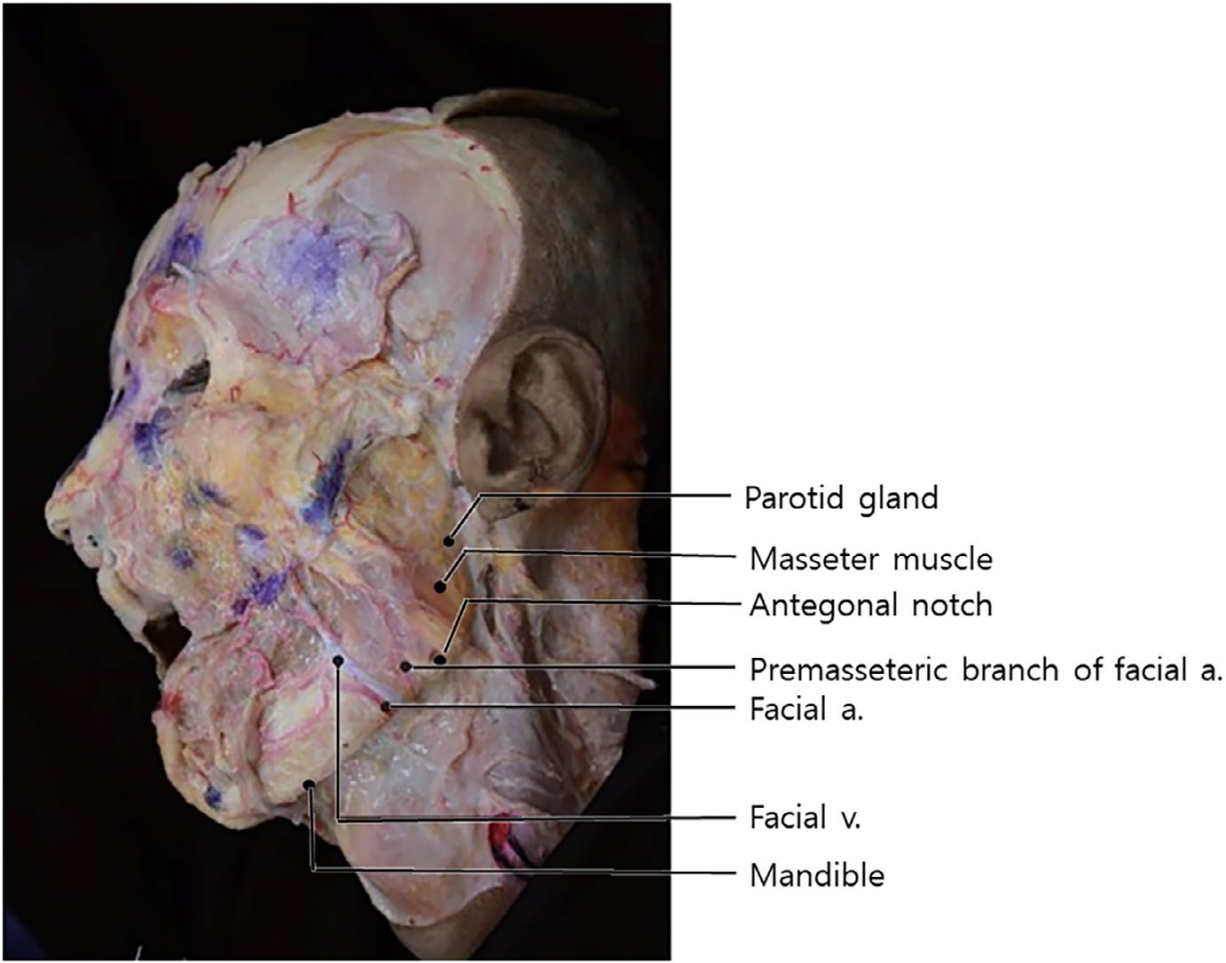
Another vessel that can cause significant bleeding is the facial artery. As previously described, the facial artery typically crosses upward between the midpoint of the inferior border of the mandible and the anterior border of the masseter muscle.

Another vessel that can cause significant bleeding is the facial artery. As previously described, the facial artery typically crosses upward between the midpoint of the inferior border of the mandible and the anterior border of the masseter muscle (Figure 3) [11].

Based on the detailed anatomical reviews provided by Lee et al. [12, 13], the facial artery's pathway is meticulously outlined, highlighting critical areas for clinical consideration. The facial artery traverses through the anterior portion of the submandibular gland, courses anterior to the masseter muscle and facial vein, and then passes through the antigonial notch before proceeding along a tortuous route. A particularly critical aspect discussed is the exposed segment of the facial artery, which is located laterally from the modiolus, typically between the zygomaticus major and risorius muscles. This segment is especially susceptible to injury due to its superficial placement, lacking significant muscle coverage, which is crucial for procedures involving the nasolabial fold.

As the facial artery passes through the area near the mandible, it is located deep within the tissues, making thread lifting procedures in the superficial fat layer around the jowls relatively safe. After ascending, the facial artery takes a complex path; it turns near the corners of the mouth, traversing above and below the facial expression muscles. It then crosses both inside and outside the nasolabial folds, branching off toward the nose and lips [14, 15].

The terminal portion of the facial artery, known as the angular artery, traverses between the subcutaneous fat layer and the muscle layer, making it unpredictable whether it runs above or below the SMAS layer. Damaging this artery can cause severe bleeding. While the zygomatic bone in the temple area allows for some degree of hemostasis through compression during bleeding, if a branch of the facial artery in the cheek area is ruptured, it is challenging to apply sufficient pressure because the blood may swell and leak into the inside of the mouth. Such an incident can lead to a substantial hematoma, causing the patient to suffer from severe bruising and swelling for two weeks to a month. Therefore, when performing thread lifting procedures that pass through the cheek area, it is crucial to handle the area gently to avoid puncturing the branches of the facial artery [15].

## 3 Nerves

The facial nerve, also known as cranial nerve VII, has five main branches that innervate various muscles of the face. The temporal branch innervates the muscles of the forehead and upper face, including the frontalis, orbicularis oculi, and corrugator supercilii muscles. The zygomatic branch supplies the muscles around the eye and upper cheek, such as the orbicularis oculi (lateral part) and some fibers of the zygomaticus major muscle. The buccal branch innervates the muscles in the midface, including the orbicularis oris, buccinator, and zygomaticus muscles, which are crucial for movements such as smiling and chewing. The marginal mandibular branch controls the muscles of the lower lip and chin, including the depressor anguli oris, depressor labii inferioris, and mentalis muscles. Lastly, the cervical branch innervates the platysma muscle, which is involved in depressing the mandible and tensing the skin of the neck. The five branches of the facial nerve, which predominantly course within the subSMAS layer in the lateral part of the face. As these nerves proceed medially, they become more superficial, increasing the risk of injury during facial procedures. To avoid potential nerve damage, it is recommended that thread lifting procedures be conducted in the supraSMAS layer.

**FIGURE 4:**
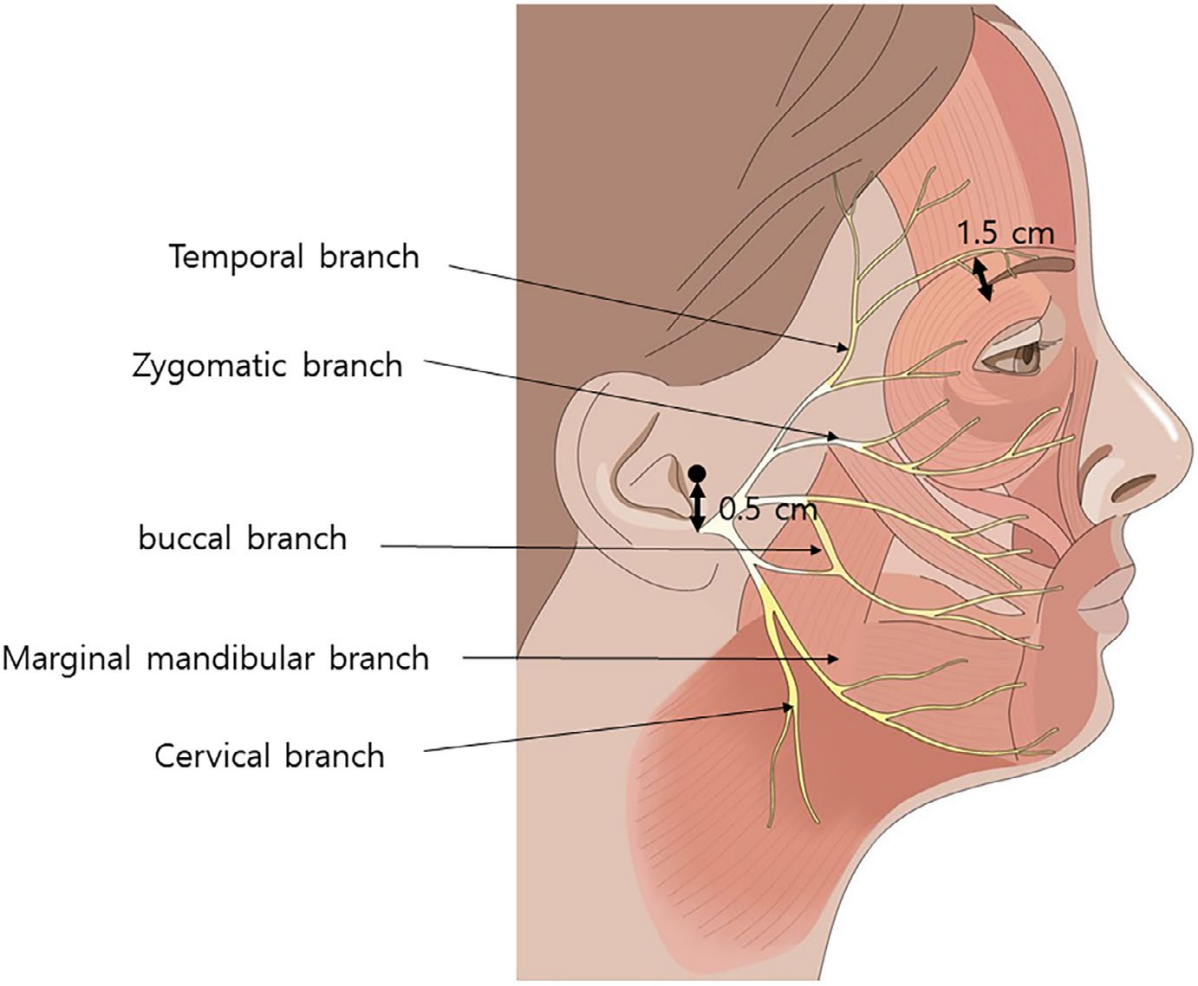
According to the Pitanguy line, the temporal branch of the facial nerve is typically located along a line extending from 0.5 cm below the tragus to 1.5 cm lateral to the eyebrow. Therefore, by ensuring gentle handling of the cannula along this line, the risk of nerve damage from the blunt end of the cannula is minimized.

Thread lifting commonly uses blunt cannulas or small needles, which significantly reduces the likelihood of severing nerves. However, care must still be taken to avoid damaging the temporal branch of the facial nerve, particularly during procedures involving thicker barbed threads in the temple area where these threads are anchored. According to the Pitanguy line, the temporal branch of the facial nerve is typically located along a line extending from 0.5 cm below the tragus to 1.5 cm lateral to the eyebrow. Therefore, by ensuring gentle handling of the cannula along this line, the risk of nerve damage from the blunt end of the cannula is minimized (Figure 4) [16]. In reality, the critical factor is not the superficial location where the nerve runs, but rather the depth at which it is situated. Therefore, when performing thread lifting procedures, it is essential to meticulously control the depth of insertion to prevent damage to the facial nerve, which could lead to impairments such as difficulty in elevating the eyebrows. Fortunately, the temporal branch of the facial nerve is not composed of a single filament; instead, it consists of 2–3 significant branches. Thus, if the cannula is manipulated gently, the likelihood of simultaneously damaging multiple nerve branches is substantially reduced. This careful approach significantly minimizes the risk of nerve injury [17].

The most common discomfort related to nerves after thread lifting is likely pain caused by the stimulation of sensory nerves when moving the mouth or the perioral area. Although this will be elaborated further in the subsequent section on space, if the threads catch around the muscles that facilitate mouth movements, pain can be felt each time the mouth is moved. Typically, this discomfort is most intense for about a week after the procedure, with gradual improvement observed over the following one to two weeks [18]. Patients occasionally report pain up to a month after the procedure, so it is advisable to provide a thorough explanation about the potential for pain, especially when using very thick threads or cogged threads, which have larger barbs. However, such pain rarely results from direct damage to the sensory nerves themselves. More commonly, if threads are placed in areas like around the mouth that frequently move, discomfort can occur even with smaller barbs. This discomfort tends to persist until the area around the caught barbs softens and adapts to the threads.

## 4 Retaining Ligaments

**FIGURE 5:**
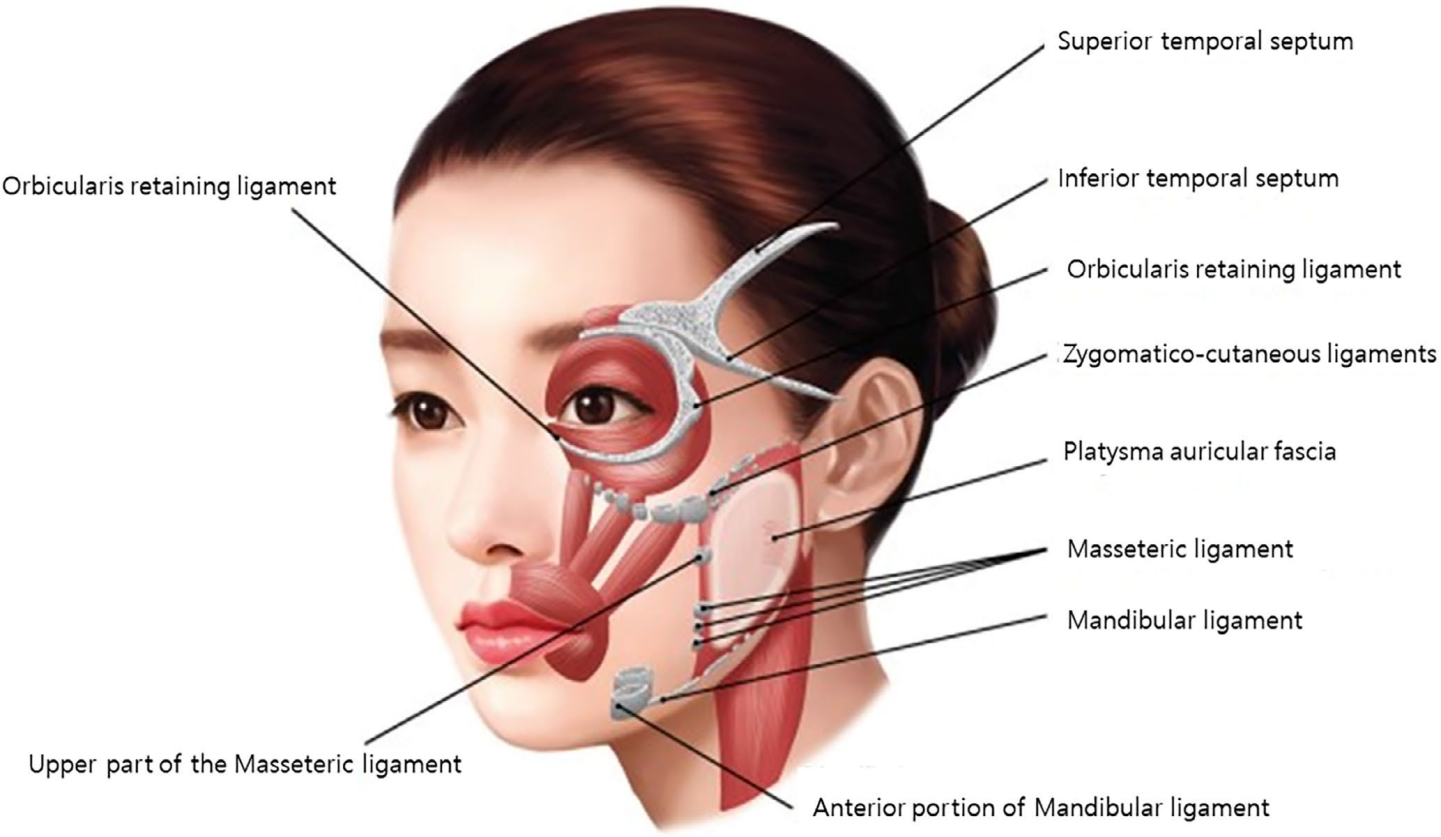
The retaining ligaments of the face, which maintain the structure of the skin and soft tissues, are traditionally classified into two types. The first type, known as true retaining ligaments, originates from the bone and extends to the skin. Examples typically considered true retaining ligaments include the orbital retaining ligament, zygomatic ligament, maxillary ligament, and mandibular ligament. The second type, known as false retaining ligaments, begins in intermediate soft tissues such as muscles or fat layers.

The retaining ligaments of the face, which maintain the structure of the skin and soft tissues, are traditionally classified into two types. The first type, known as true retaining ligaments, originates from the bone and extends to the skin. Examples typically considered true retaining ligaments include the orbital retaining ligament, zygomatic ligament, maxillary ligament, and mandibular ligament. The second type, known as false retaining ligaments, begins in intermediate soft tissues such as muscles or fat layers. These classifications help in understanding the different structural supports within the facial anatomy and are used to differentiate the ligaments based on their attachments and anatomical functions (Figure 5) [19].

The description of the facial retaining ligaments is intriguing. These structures function akin to actual ligaments, connecting the skin and soft tissues to bones in certain areas of the face. Unlike typical ligaments in the body, these are not viewed as true ligamentous tissues but rather as dense clusters of soft tissue that firmly anchor the skin and soft tissues, preventing sagging. This adaptation is crucial for humans, who need to exhibit a wide range of facial expressions. The face is uniquely designed so that not all skin and soft tissues are directly attached to the bones, allowing for movement necessary for expression and speech. The presence of these pseudo‐ligamentous tissues differentiates areas that need to be mobile from those that require strong attachment to the bones.

In aesthetic procedures like thread lifting, the importance of these facial retaining structures becomes evident. Even when using non‐fixed threads, the robust nature of these pseudo‐ligaments plays a vital role in anchoring the threads in place, ensuring the desired aesthetic effect is maintained [20].

In the context of thread lifting procedures targeting the midface, the zygomatic and masseteric cutaneous ligaments are particularly crucial. These two retaining ligaments intersect in a T‐shape, providing robust and strong support for the skin and soft tissues. Such structural support is essential for the efficacy of thread lifts, particularly when using non‐bidirectional, zigzag type multidirectional cog threads.

**FIGURE 6:**
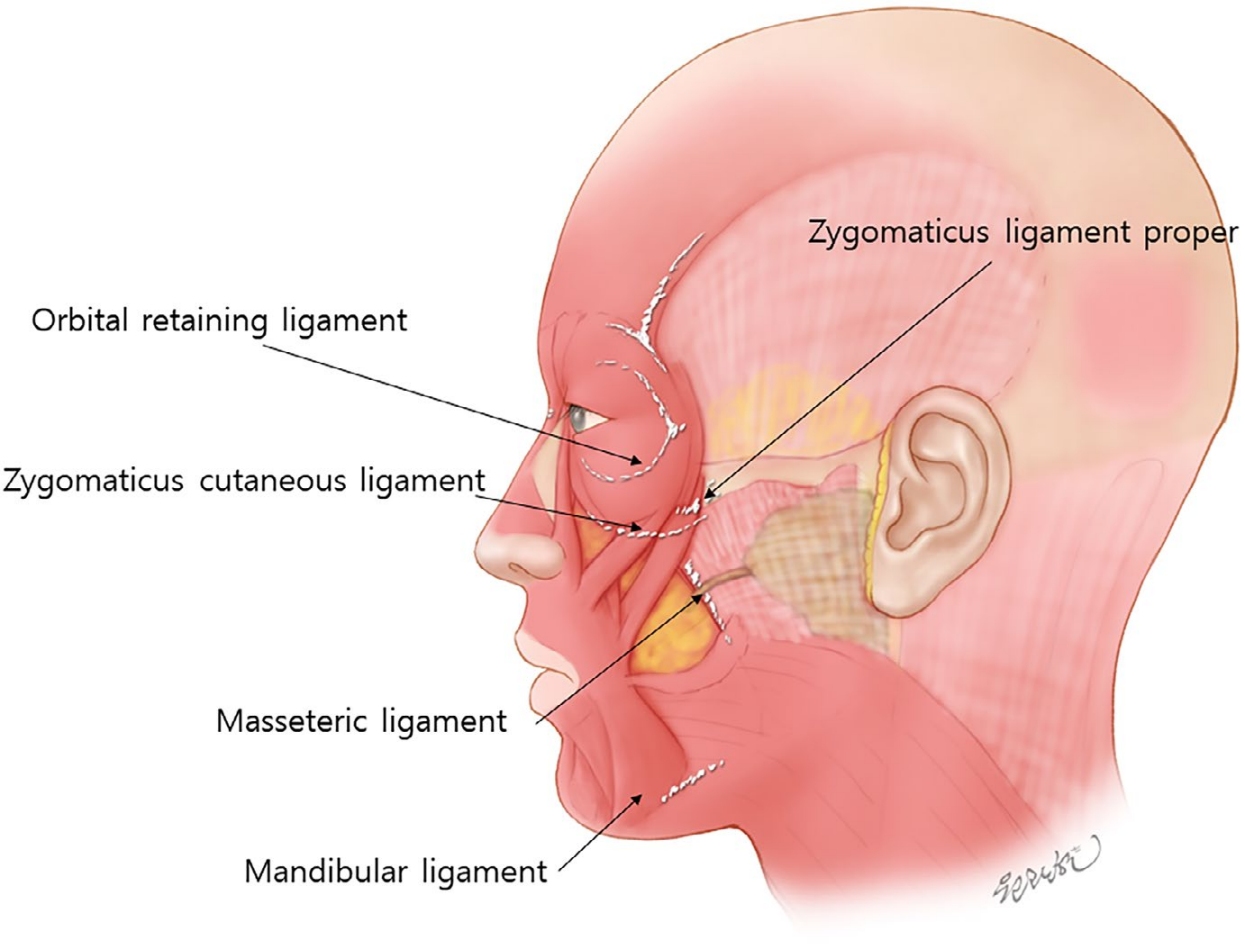
When these types of threads are anchored in these dense ligamentous tissues, they effectively engage the underlying soft tissues intended to be lifted. Even without additional anchoring points above, the threads caught within these ligaments can create a lifting effect, as if they were anchored further up. This leverages the natural strength and positioning of the ligaments to maintain the lift, showcasing the critical role of anatomical understanding in optimizing aesthetic outcomes in facial thread lifting procedures.

When these types of threads are anchored in these dense ligamentous tissues, they effectively engage the underlying soft tissues intended to be lifted. Even without additional anchoring points above, the threads caught within these ligaments can create a lifting effect, as if they were anchored further up. This leverages the natural strength and positioning of the ligaments to maintain the lift, showcasing the critical role of anatomical understanding in optimizing aesthetic outcomes in facial thread lifting procedures (Figure 6) [21]. However, there is a cautionary aspect to consider with the use of these retaining ligaments in thread lifting procedures. If the threads are overly anchored within these ligaments, they can create persistent dimpling of the skin and soft tissues, which are difficult to resolve even with significant effort. This potential complication necessitates careful placement of the threads.

Particularly, if the ends of the threads catch on the maxillary or mandibular ligaments—which are located in the lower parts of the face—it can also lead to dimpling below these areas. Post‐procedure, it is crucial to examine these regions to ensure that the barbs of the threads are not excessively engaging the ligamentous tissues. If severe dimpling occurs due to the threads being too tightly caught, early intervention with massage techniques is recommended to alleviate the tension and smooth out the dimples. This proactive approach helps to optimize the aesthetic outcomes while minimizing unwanted textural changes to the skin [22].

### 4.1 Line of Ligament

Casabona et al. [23] defined the “line of ligaments” serves as a crucial guide in thread lifting procedures, especially in determining optimal anchoring points. Running vertically just lateral to the lateral orbital rim, this line extends from the temporal crest to the mandible and connects key facial ligaments such as the temporal ligamentous adhesion, lateral orbital thickening, zygomatic ligament (McGregor's patch), and mandibular ligament. The facial structures lateral to this line is more fixed, providing a stable foundation ideal for anchoring and fixing threads. This stability is essential for creating a lifting effect, as the threads can effectively reposition and secure the facial tissues. In contrast, the medial facial regions are relatively more mobile and less suitable for anchoring, but they benefit from the lift achieved by repositioning the more fixed lateral tissues. Utilizing the lateral face as a primary anchoring point in thread lifting procedures not only enhances the effectiveness of the lift but also reduces the risk of complications, ensuring better aesthetic outcomes.

### 4.2 Nomenclature of the Ligaments

The nomenclature of facial ligaments has been a topic of debate and confusion within the medical community, largely due to varying interpretations and terminologies used across different studies. The term “retaining ligament” was first introduced by Dr. McGregor, later officially named “McGregor's patch” by Kaye, which refers to the fibrous attachment area in the cheek region. Furnas expanded this concept by detailing structures like the zygomatic ligament and masseteric ligament, which connect the facial skin to the underlying skeletal structures. These ligaments, also referred to as subcutaneous ligamentous attachments, are critical for maintaining facial contour and structure. The nomenclature is further complicated by differences in describing the locations and extents of these ligaments, particularly for the zygomatic and masseteric ligaments. Terms like “platysma auricular ligament,” “parotid cutaneous ligament,” and “temporoparotid fascia” have been used interchangeably in the literature, leading to confusion. The variability in descriptions often arises from different anatomical studies, cadaver dissections, and clinical observations, making a unified classification challenging. Understanding these ligaments' precise anatomy and nomenclature is essential, especially for aesthetic and reconstructive procedures involving the face [24].

## 5 Fat Compartments

As we age, the elasticity of our skin and soft tissues diminishes, which in conjunction with gravity, leads to the sagging of fat tissues. Traditionally, it was believed that these fat tissues were interconnected as a single unit, causing them to sag uniformly. However, anatomical studies have revealed that fat tissues actually shift downward within their distinct fat compartments. This finding indicates that the sagging occurs compartmentally, rather than uniformly across all facial fat.

This compartmental movement is consistent across both superficial and deep layers of fat tissue. This insight has significant implications for aesthetic treatments, including the strategic placement of fillers and the use of lifting techniques. Understanding that fat compartments shift independently allows clinicians to target specific areas more effectively, tailoring interventions to the unique structural changes that occur with aging. This approach can lead to more natural and harmonious aesthetic outcomes [25].

When planning a thread lifting procedure, it is essential to carefully consider the relationships between fat tissues, skin, and retaining ligaments to set specific goals. Typically, the primary targets for elevation in thread lifting are the superficial fat compartments. For instance, the nasolabial fat, which exacerbates the appearance of smile lines, and the superior and inferior jowl fats, which contribute to jowling and marionette lines, are common areas that are lifted during the procedure.

**FIGURE 7:**
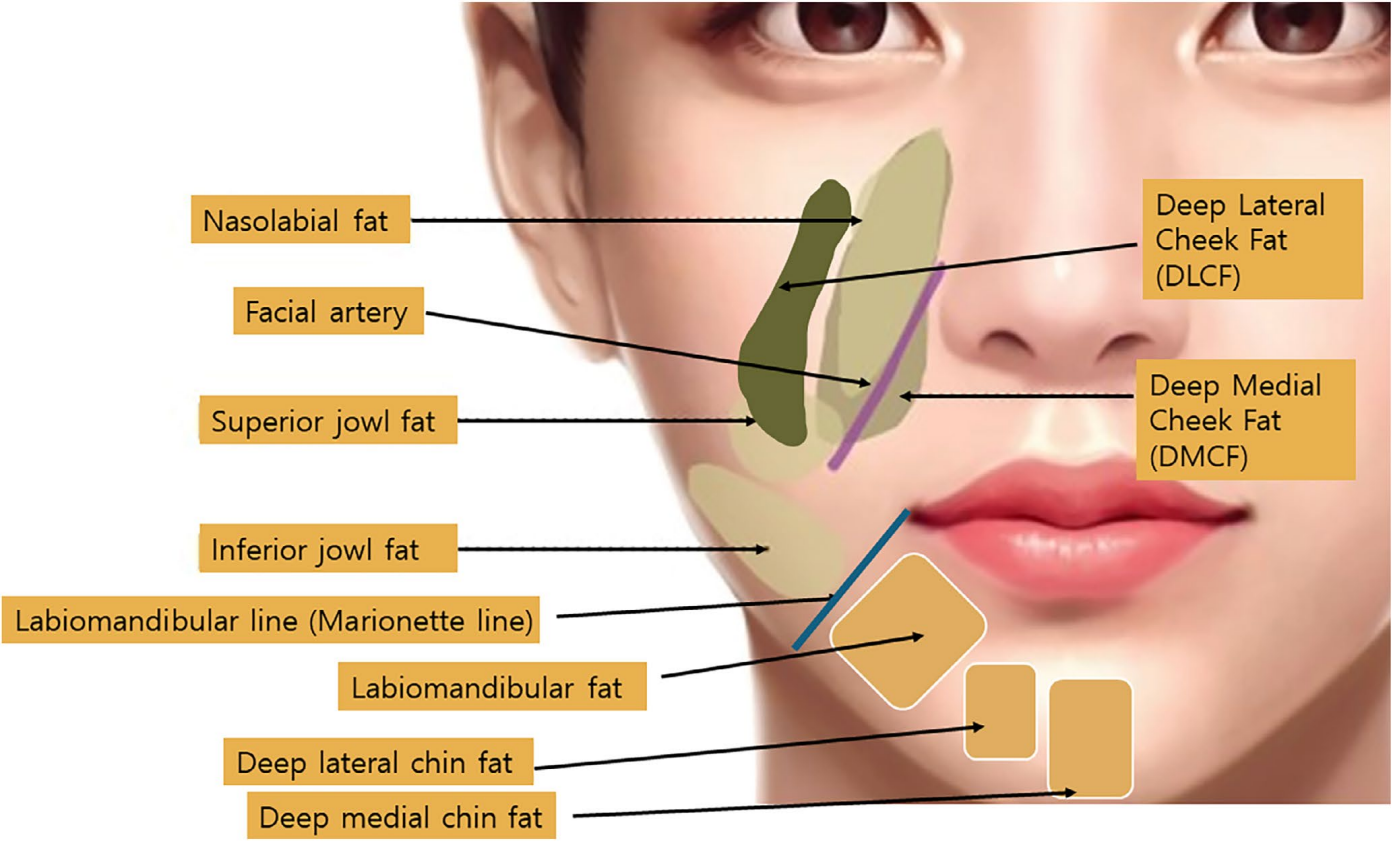
The strategic targeting of these specific fat compartments allows for a more focused and effective lifting effect, helping to counteract the sagging and restore a more youthful facial contour. By understanding the anatomy and how each compartment contributes to overall facial aging, clinicians can use thread lifting to achieve more precise and visually pleasing results. This tailored approach ensures that the lifting addresses the most impactful areas, enhancing the natural features of the face. Medial to the marionette line is the labiomandibular fat, which typically loses volume with age, accentuating the appearance of marionette lines. Lateral to the marionette line are the superior and inferior jowl fat areas, which tend to increase in volume with age, further deepening these lines.

The strategic targeting of these specific fat compartments allows for a more focused and effective lifting effect, helping to counteract the sagging and restore a more youthful facial contour. By understanding the anatomy and how each compartment contributes to overall facial aging, clinicians can use thread lifting to achieve more precise and visually pleasing results. This tailored approach ensures that the lifting addresses the most impactful areas, enhancing the natural features of the face (Figure 7) [26].

The development of nasolabial folds, commonly referred to as smile lines, can be attributed to various factors that interact in complex ways, but one key element associated with their progression as we age is the change in volume of fat tissues. Anatomical studies have indicated that the superficial fat compartments located above the SMAS (Superficial Musculoaponeurotic System) layer tend to increase in volume with age, while the deep fat compartments beneath it tend to decrease in volume.

**FIGURE 8:**
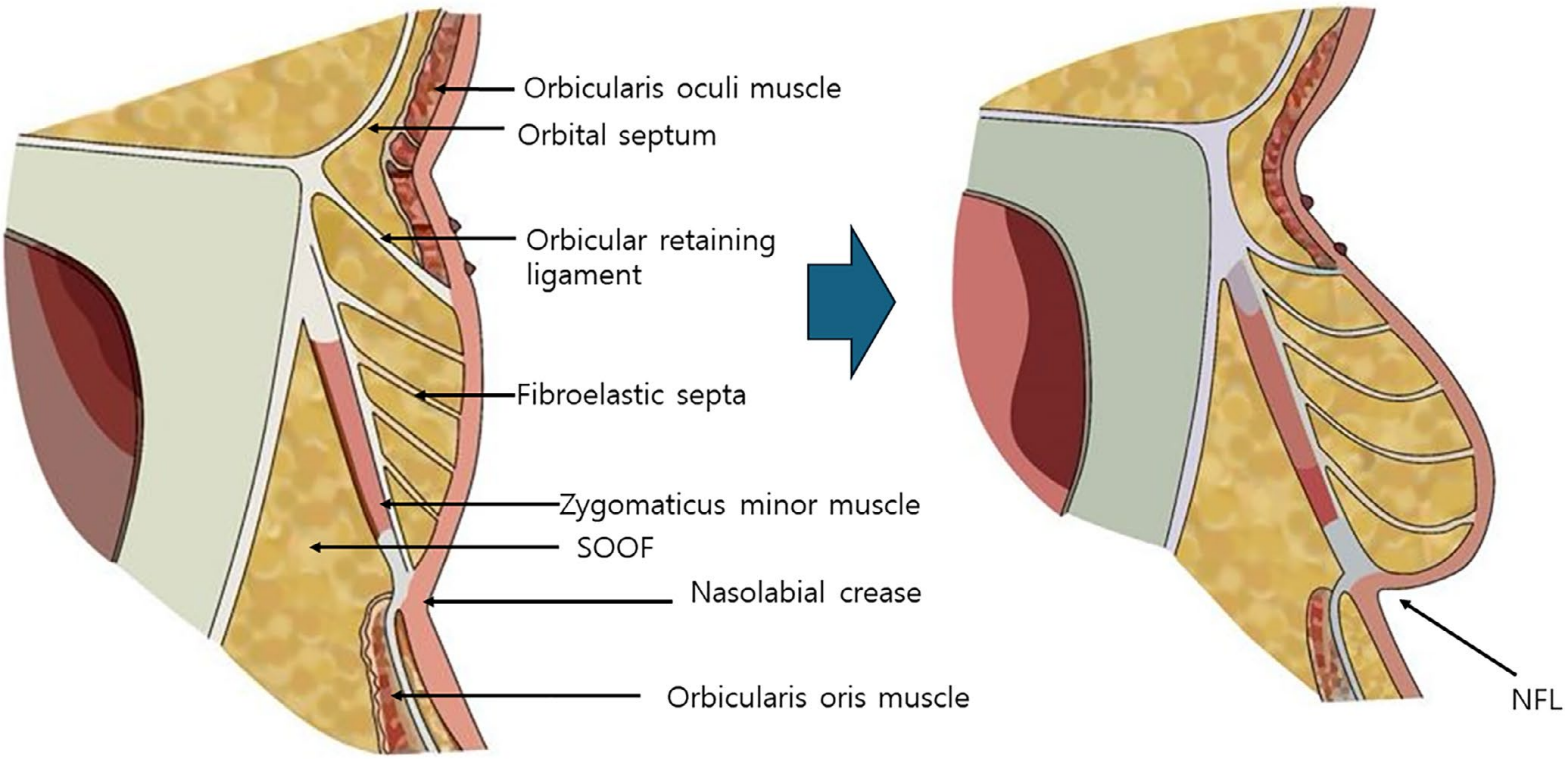
Specifically, in the area of the nasolabial folds, the nasolabial fat, which is a type of superficial fat, increases in volume and sags downward. In contrast, the deep medial cheek fat (DMCF), positioned deeper and below the nasolabial fat, experiences a decrease in volume and also sags downward. This differential volume change contributes significantly to the deepening of the nasolabial folds. The increase in superficial fat pushes the skin outward and downward, while the decrease in deep fat volume creates a lack of underlying support, exacerbating the sagging and formation of deeper folds.

Specifically, in the area of the nasolabial folds, the nasolabial fat, which is a type of superficial fat, increases in volume and sags downward. In contrast, the deep medial cheek fat (DMCF), positioned deeper and below the nasolabial fat, experiences a decrease in volume and also sags downward. This differential volume change contributes significantly to the deepening of the nasolabial folds. The increase in superficial fat pushes the skin outward and downward, while the decrease in deep fat volume creates a lack of underlying support, exacerbating the sagging and formation of deeper folds. This understanding is crucial for effectively targeting treatments, such as fillers or thread lifts, to appropriately support and volumize these areas to mitigate the appearance of aging (Figure 8) [27, 28].

As the volume of the deep medial cheek fat (DMCF) decreases, the sagging of the deep fat layer is less noticeable compared to the increased sagging of the expanded superficial fat layer, including the nasolabial fat. This dynamic exacerbates the appearance of nasolabial folds, making them appear deeper.

Taking this mechanism into account, a combination treatment approach is recommended to effectively address the deepening of nasolabial folds due to aging. This would involve:

### 5.1 Filler Injection

Injecting fillers into the deeper layers beneath the SMAS, specifically in the sunken areas medial to the nasolabial fold line, can help restore volume. This provides support to the upper soft tissues that have lost their foundational volume due to the reduction in the deep fat compartments.

### 5.2 Thread Lifting

For the sagging tissues lateral to the nasolabial fold line, a thread lift targeting the superficial fat layer above the SMAS can be used. This helps in lifting and repositioning the sagging superficial fat, reducing the severity of the folds and improving the overall facial contour.

**FIGURE 9:**
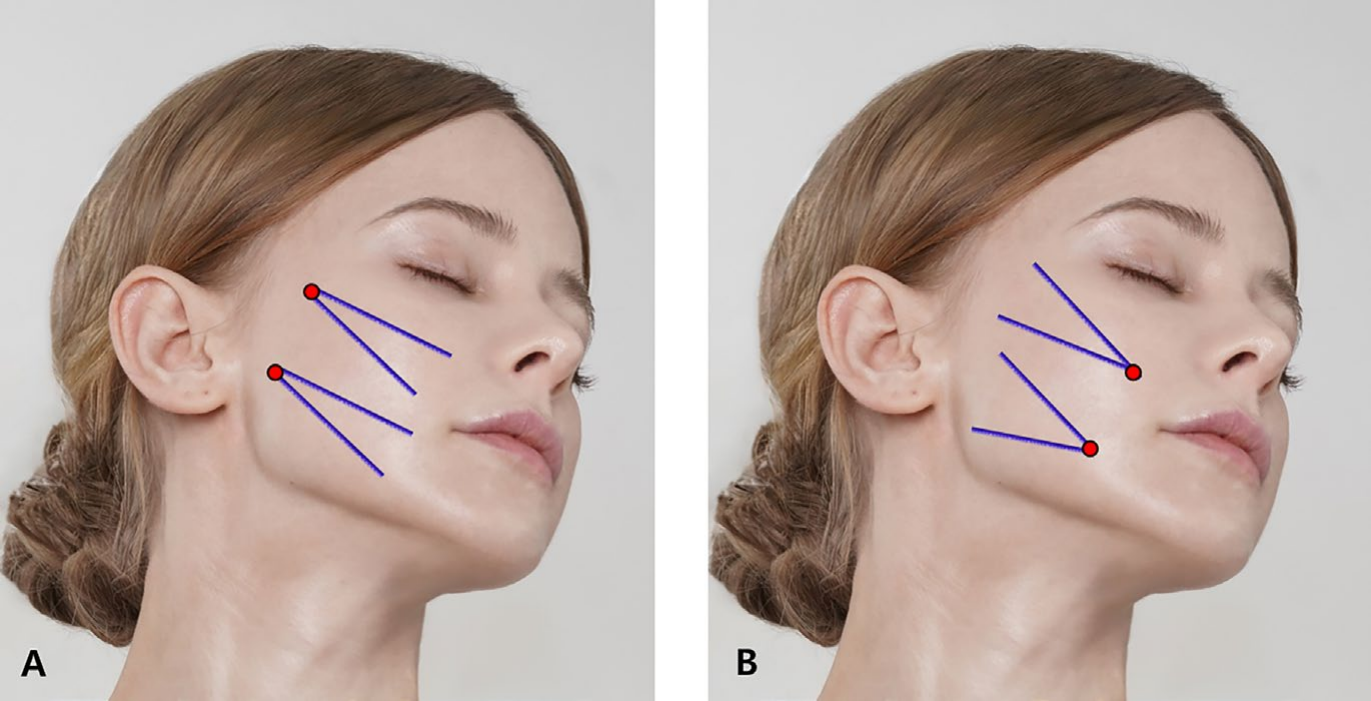
The nasolabial and marionette line correction can be performed using antegrade (A) and reverse (B) vectors of bidirectional thread insertion. The threads used are 7 cm long Secret Illusion threads and LVDR (Hyundaemeditech Inc., Wonju, Republic of Korea and Sihler Inc., Seoul, Republic of Korea).

This combination treatment approach ensures that both the loss of volume and the excess sagging are addressed, offering a more comprehensive and balanced rejuvenation. By correcting volume deficits deep within the facial structure and providing lift to the more superficial sagging tissues, the depth and visibility of nasolabial folds can be significantly reduced [27]. In addition to nasolabial folds, marionette lines are another common concern addressed during thread lifting, which often become more pronounced with age due to sagging cheeks and the area around the mouth. Marionette lines run vertically from the corners of the mouth toward the chin, and the anatomy around these lines is critical to understanding how to effectively treat them. Nasolabial and marionette line correction can be performed using antegrade and reverse vectors of bidirectional thread insertion (Figure 9).

### 5.3 Anatomical Background

#### 5.3.1 Labiomandibular Fat

Located medially to the marionette line, this fat compartment typically loses volume as one ages. This volume loss can create a hollow appearance, making the marionette lines appear more pronounced.

Superior and inferior jowl fat: Situated laterally to the marionette line, these areas of fat tend to increase in volume with age. The increase in volume here can exacerbate the sagging and deepening of the marionette lines, as the added weight pulls down the skin further.

#### 5.3.2 Treatment Strategy

To effectively address marionette lines, a targeted approach is essential:

#### 5.3.3 Filler Injection

Injecting dermal fillers into the labiomandibular fat area can help to restore lost volume and provide support to the skin, potentially reducing the appearance of the lines by smoothing out the area.

#### 5.3.4 Thread Lifting

For the jowl fats that have increased in volume and sagged, a thread lift can reposition these fats upward, tightening the skin and lessening the depth of the marionette lines. Threads can be strategically placed to lift the sagging tissues and provide a more youthful contour to the lower face.

This combined approach not only addresses the structural changes due to volume loss and gain in specific areas but also offers a way to rejuvenate the lower face holistically, providing balance and natural‐looking results. By understanding the specific anatomical changes and targeting each area appropriately, the effects of aging around the marionette lines can be effectively mitigated (Figure 7) [29].

The strategy of using a combination of thread lifting and filler treatments is similarly beneficial for addressing marionette lines, as it is for correcting nasolabial folds. For marionette lines:

#### 5.3.5 Thread Lifting

This technique is used to lift the sagging tissues that have increased in volume and are located laterally to the marionette lines. By repositioning these tissues upward, the appearance of sagging and the depth of the lines can be reduced.

#### 5.3.6 Filler Treatment

Fillers are applied medially to the marionette lines where the fat has decreased, causing hollows. By restoring volume in these areas, the skin appears smoother and the depth of the marionette lines is lessened.

Regarding the lifting of buccal fat with thread lifting, there are varying opinions in the medical community. Buccal fat, which is about 10 cc in volume, is not only situated in the cheek but also extends upward toward the zygomatic arch. The portion of the buccal fat that lies under the zygomatic arch is known as the temporal extension of the buccal fat, or the deep temporal fat pad.

The anatomy and distribution of buccal fat are significant because they influence the potential outcomes of aesthetic procedures:

#### 5.3.7 Buccal Fat's Role in Facial Aesthetics

The buccal fat pad plays a crucial role in defining midface contours. Excessive removal or inappropriate manipulation can result in an overly gaunt appearance, while conservative and strategic treatment can enhance facial harmony.

#### 5.3.8 Treatment Considerations

Given the interconnected nature of buccal and temporal fat, clinicians must carefully assess the individual's facial structure and fat distribution before deciding on a lifting strategy. The decision to lift or modify this fat should be tailored to achieve balance and natural‐looking results while avoiding an unnatural hollow appearance.

Each treatment plan should be customized based on the detailed anatomical understanding and patient‐specific facial features to ensure the most effective and aesthetically pleasing outcome [30].

Buccal fat serves to reduce friction among perioral tissues during actions such as sucking in infancy and provides protection for vital vascular and neural structures surrounding the mouth. As individuals age and increasingly utilize the masseter muscle, the role of buccal fat diminishes. Typically, the volume of this fat continues to increase until around the age of 50, after which it begins to decrease—a pattern similar to that observed in the deep fat compartments, which also reduce in volume with age. These fat tissues, located deep beneath the zygomatic arch, support the skin and soft tissues above. Inadequate support from these tissues can lead to the development of sunken cheeks [31].

As buccal fat volume decreases, it can lead to the appearance of sunken cheeks and sagging. Consequently, one might consider that lifting the buccal fat through thread lifting could simultaneously improve both the hollowness and sagging. However, it is crucial to note that buccal fat is situated deeper than the SMAS layer and contains several important anatomical structures, including blood vessels, branches of the facial nerve, and the parotid duct. Directly targeting the buccal fat with needles poses a risk of damaging these structures, potentially leading to severe complications, such as inadvertently puncturing the nearby parotid gland.

Moreover, realistically considering the risks, even if thread lifting is performed, buccal fat, unlike other fat tissues, contains fewer firm fibers and is more gelatinous, which means that the barbs of the threads may not effectively engage, leading to suboptimal lifting effects or difficulty in maintaining the lift.

Therefore, in such cases, it is more advisable to target the capsule tissue surrounding the buccal fat rather than the buccal fat itself for thread lifting. This approach minimizes the risk of damaging critical anatomical structures and may offer a more stable and effective lifting outcome [32].

## 6 Space

The term “facial space” refers to the anatomically demarcated regions within the face, bounded by layers such as fat, SMAS (superficial musculoaponeurotic system), fascia tissues, muscles, and retaining ligaments, although no physical tissue envelops these spaces explicitly. These spaces typically form between the superficial and deep fascial layers, facilitating independent movement of various facial expression muscles without interfering with each other. For example, the orbicularis oculi muscle and orbicularis oris muscle can operate independently from the zygomaticus major and minor muscles, which elevate the corners of the mouth. This allows each muscle to contract freely without affecting the other.

Moreover, blood vessels and nerves often run along the boundaries of these facial spaces, which minimally contain critical structures within their confines. This anatomical feature makes these spaces safer pathways for surgical dissection or for the safe insertion and passage of needles or cannulas during procedures. In the context of thread lifting, especially when using thicker cogged threads, these facial spaces provide a smooth pathway for the cannula and threads to pass through, enhancing the safety and efficacy of the procedure. This strategic utilization of facial spaces is crucial for minimizing the risk of damaging important anatomical structures during aesthetic enhancements [33].

While facial spaces facilitate certain aspects of thread lifting by providing clear pathways and allowing for independent muscle movements, they also pose potential challenges. As these spaces act as gliding planes for facial expression muscles, inserting thick threads with barbs, especially around areas of frequent movement like the perioral region, can provoke discomfort or pain.

One particular area that requires careful consideration is the premasseteric space, which lies superficial to the masseter muscle. This space is anatomically bordered anteriorly by the masseteric cutaneous ligament, superficially by the platysma muscle, inferiorly by the masseter muscle, and below by the mandibular ligament and septum. Given its location and the structures it contains, the premasseteric space is highly sensitive. Introducing thick, barbed threads into this area can cause significant discomfort due to the density and sensitivity of the surrounding structures.

**FIGURE 10:**
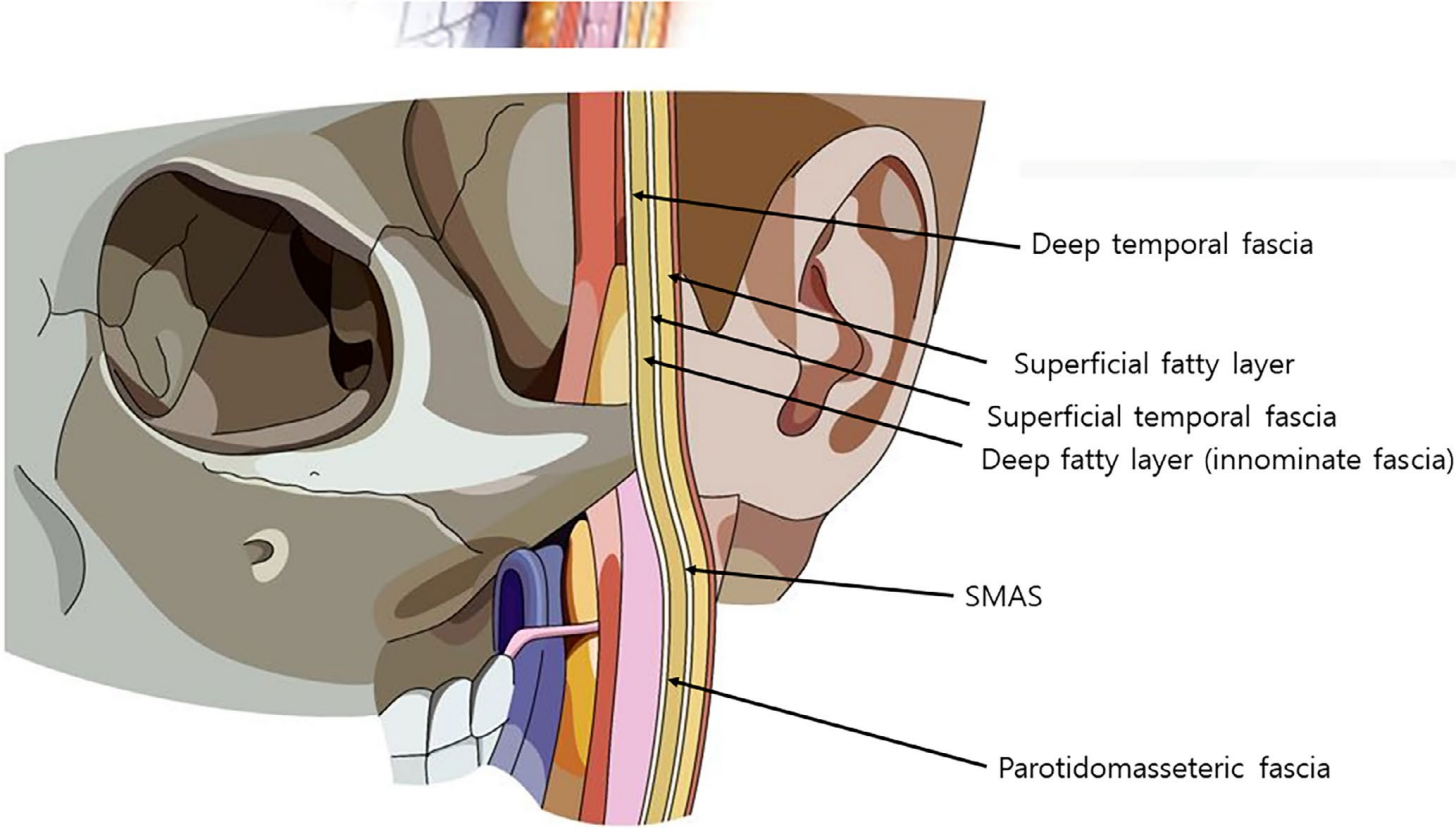
Therefore, when performing thread lifts, it is crucial to evaluate the specific characteristics and sensitivities of each facial space. Understanding these nuances ensures that the procedure minimizes discomfort and maximizes effectiveness, particularly in areas prone to frequent movement and where important anatomical structures converge.

Therefore, when performing thread lifts, it is crucial to evaluate the specific characteristics and sensitivities of each facial space. Understanding these nuances ensures that the procedure minimizes discomfort and maximizes effectiveness, particularly in areas prone to frequent movement and where important anatomical structures converge (Figure 10) [34].

When threads are inserted into such spaces, their entry may feel particularly smooth due to the minimal resistance encountered. This can give practitioners the impression that the procedure is proceeding smoothly and effortlessly, as there is no resistance from the tissue. However, while this ease of insertion can seem advantageous from the practitioner's perspective, it comes with potential downsides.

If the threads are placed too close to the masseter muscle within the premasseteric space, the patient might experience significant discomfort or pain during activities involving the mouth, such as speaking, moving the jaw, or chewing. This discomfort arises because the masseter muscle is one of the primary muscles involved in mastication and jaw movement. The presence of a foreign object like a thread near this actively moving muscle can lead to irritation or pain, particularly when the muscle contracts during these activities.

Therefore, while the low resistance of certain facial spaces can facilitate the ease of thread insertion, careful consideration must be given to the anatomical location and the potential impact on muscle function. Ensuring that threads are positioned at a safe distance from critical muscles like the masseter is crucial to avoid post‐procedural discomfort and to achieve a successful aesthetic outcome [34, 35].

In lower face thread lifting, it is crucial to avoid the temptation to continue threading into the noticeably looser spaces that you might encounter beneath the SMAS layer, where resistance suddenly decreases. While these areas may offer less resistance and thus seem easier for insertion, navigating the procedure in this manner can lead to suboptimal outcomes and increased risk of complications.

Instead, it is safer and more ideal to either penetrate through the tougher SMAS layer or to stay just above it, threading through the base of the superficial fat. This approach targets the desired tissue layers, such as the superior and inferior jowl fat, more precisely. By anchoring the threads' barbs within these specific layers, the practitioner can achieve effective lifting and tightening of the facial contours without the risks associated with deeper, less resistant spaces.

This method ensures that the threads are securely placed in areas that can structurally support the lift, providing both immediate and long‐lasting aesthetic improvements while minimizing the risk of discomfort or movement interference associated with deeper placement [35, 36].

Indeed, performing procedures with precision in an area that cannot be visually inspected directly, like the SMAS layer during thread lifting, can be challenging. Fortunately, the SMAS (Superficial Musculoaponeurotic System) is not a single homogeneous layer but rather comprises a mille‐feuille‐like structure of very thin, multiple layers of fibrous tissues that form an overall layer.

During thread lifting, navigating the cannula through the SMAS requires a keen sense of touch to accurately gauge the resistance offered by these layers. The practitioner can feel the texture and resistance changes as the cannula passes through these fibrous layers. Ideally, inserting the thread within or just slightly above these layers, where slight resistance is felt, allows for effective placement of the thread's barbs. This method ensures that the threads are anchored securely within the structural framework of the SMAS, enhancing the lifting effect and ensuring the longevity and stability of the results.

This tactile feedback is crucial for optimizing the placement of threads and minimizing potential risks or complications associated with incorrect depth of insertion. By mastering the feel of the different resistance levels within the SMAS, practitioners can achieve more precise and effective outcomes in thread lifting procedures [37].

## 7 Parotid and Submandibular Gland

When performing thread lifting, particular caution must be exercised regarding the salivary glands, specifically the submandibular and parotid glands. While common complications associated with thread lifting, such as bruising and swelling due to vascular injury, are typically transient and not long‐lasting, and nerve damage generally does not result in permanent sensory or motor function impairment, damage to salivary glands can lead to more serious complications.

If a thread inadvertently penetrates a salivary gland or the parotid duct, it can cause saliva to leak into the surrounding soft tissues. This leakage can result in a condition known as sialocele, where a cyst forms due to the accumulation of saliva. Sialocele is not only uncomfortable but can also be challenging to treat and may require interventions such as drainage or even surgery.

Therefore, it is crucial for practitioners to have a thorough understanding of the anatomical locations of these glands and ducts and to use meticulous techniques during thread lifting to avoid penetrating these structures. Accurate placement of threads away from these critical areas reduces the risk of such adverse effects and ensures a safer and more effective treatment outcome.

The submandibular gland is typically delineated superiorly by the zygomatic arch, posteriorly by the ear lobe, and inferiorly by the mandibular border. However, it occasionally extends beyond the posterior boundary of the mandibular ramus, causing the area beneath the ear to appear protruded. As discussed in the section on botulinum toxin, the facial nerve traverses between the superficial and deep lobes of the submandibular gland. Additionally, at the anterior boundary where the parotid duct emerges, there may also be an accessory parotid gland present [38].

**FIGURE 11:**
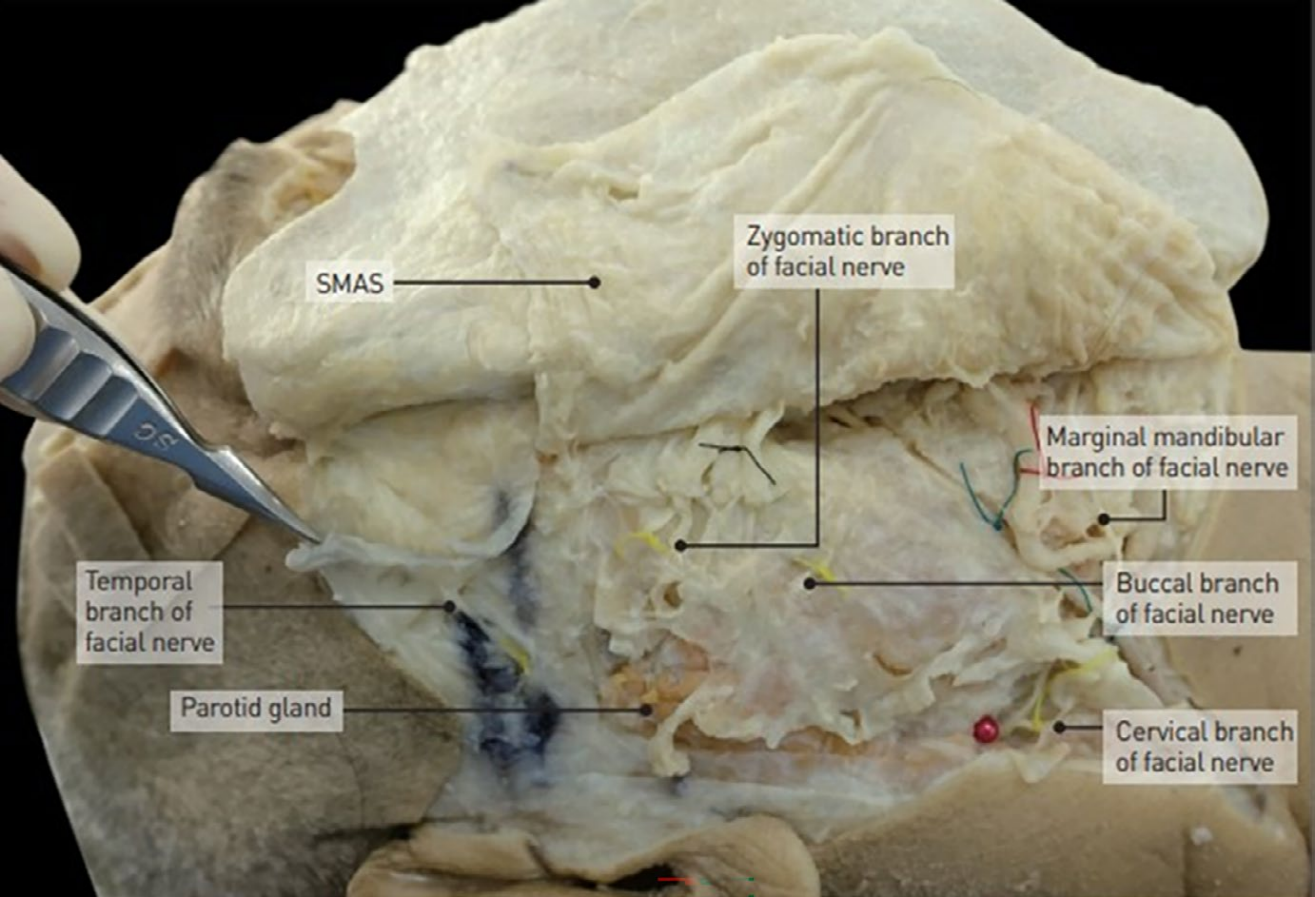
The thread should be placed above the SMAS layer since all the facial nerves runs beneath the SMAS layer.

The submandibular gland is anatomically positioned beneath the deep fascia of the face, below the layer of the Superficial Musculoaponeurotic System (SMAS). This gland is encapsulated within the parotid capsule, which is a deeper structural component. The glandular substance of the submandibular gland is enveloped within this capsule, providing a distinct anatomical layering that influences both the function and potential interventions in this region (Figure 11) [39].

Therefore, when performing thread lifting, great care must be taken not to penetrate the parotid capsule that envelops the submandibular gland substance, as it is quite dense. To avoid damaging this area, the cannula should be inserted slowly and parallel to the skin surface during thread lifting. However, during the procedure, if the patient clenches their teeth or moves due to discomfort, the submandibular gland may move closer to the skin surface, increasing the risk of injury. If there is any indication that the cannula has penetrated too deeply, potentially reaching the submandibular gland, it should be withdrawn and reinserted to ensure it is safely within the correct layer. This precaution helps to avoid injury to the gland and ensures the effectiveness and safety of the thread lifting procedure [40].

**FIGURE 12:**
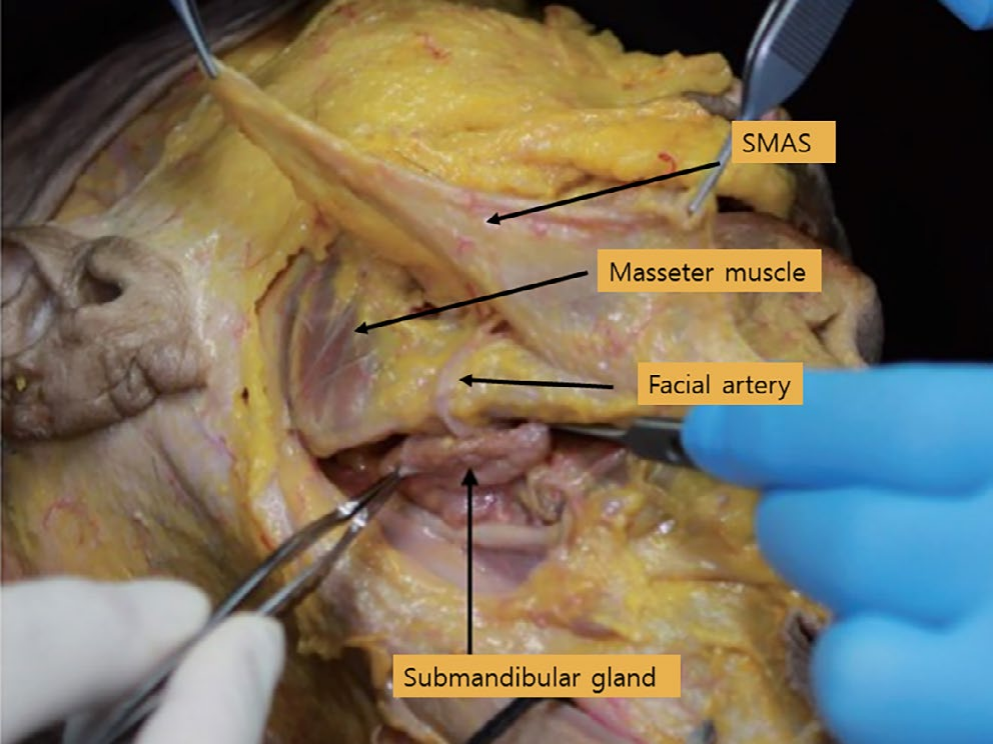
The submandibular gland is located deep within the lower boundary of the mandible, approximately within the posterior two‐thirds of the inner jawline. Due to its deep positioning, it is not easily observable visually. However, one can palpate this gland by placing a finger in the specified area and asking the patient to swallow saliva. During this action, the gland can be felt as a somewhat firm structure under the jaw. This palpation technique helps in identifying the gland's location for diagnostic or therapeutic purposes.

The submandibular gland is located deep within the lower boundary of the mandible, approximately within the posterior two‐thirds of the inner jawline. Due to its deep positioning, it is not easily observable visually. However, one can palpate this gland by placing a finger in the specified area and asking the patient to swallow saliva. During this action, the gland can be felt as a somewhat firm structure under the jaw. This palpation technique helps in identifying the gland's location for diagnostic or therapeutic purposes (Figure 12) [41].

During the correction of a double chin using a cannula, passing beneath the platysma muscle through the softer layers without penetrating too deeply is essential to minimize the risk of puncturing the submandibular gland. The submandibular gland, located in the lower jaw area, can be more prominent in patients with little fat and thin skin in this region. In cases where patients mistakenly attribute the appearance of a protruding submandibular gland to a double chin, they may seek thread lifting as a solution. However, hypertrophy of the submandibular gland cannot be corrected through thread lifting, and attempting to do so may inadvertently cause damage to the gland.

Therefore, for such patients, it is crucial to conduct an accurate diagnosis to clarify that the prominence is due to glandular hypertrophy and not excess fat. For these cases, alternative treatments like salivary gland Botox, which targets the gland to reduce its size and appearance, should be recommended rather than thread lifting. This approach ensures that the actual condition is treated appropriately without unnecessary risks [42].

## 8 Discussion

Thread lifting stands out as a procedure that not only offers visible lifting effects but also requires intricate knowledge of facial anatomy for safe and successful outcomes. A critical aspect of this knowledge pertains to the vascular anatomy of the face. Practitioners must have the ability to identify and strategically avoid significant blood vessels such as the superficial temporal artery (STA) and the facial artery. Successfully navigating these vascular structures is paramount to prevent complications such as excessive bleeding, which can be severed and required immediate intervention. Mastery in this area not only enhances the safety of the procedure but also minimizes the risk of post‐procedure bruising and swelling, thereby improving patient recovery time and satisfaction.

Equally important in thread lifting is the understanding of neural pathways, specifically the branches of the facial nerve. Avoiding nerve damage is crucial, as inadvertent injury can lead to temporary or even permanent facial paralysis. Utilizing blunt cannulas and adhering to known safe zones identified through anatomical studies helps mitigate this risk. By reducing the likelihood of nerve damage, clinicians can preserve facial functionality while achieving desired aesthetic enhancements. This careful navigation ensures that while the physical appearance is enhanced, the functional integrity of the facial muscles remains intact, maintaining natural expressions and movements.

Another fundamental element in thread lifting is the strategic manipulation of facial fat compartments. As the face ages, the distribution and volume of facial fat change, often leading to sagging and the formation of deep folds and wrinkles. Thread lifting allows for precise adjustments and repositioning of both superficial and deep fat compartments. This targeted approach not only helps in lifting the sagging areas but also helps in redefining facial contours to a more youthful state. Understanding how these compartments shift with age enables practitioners to tailor their techniques to the individual's specific aging pattern, resulting in more natural and harmonious aesthetic outcomes.

The use of real‐time imaging techniques such as ultrasound has become increasingly valuable in ensuring the safety and precision of facial thread lifting procedures. According to Yi et al. [43], ultrasonography aids in accurately visualizing underlying anatomical structures, such as nerves and blood vessels, thereby minimizing the risk of complications like neurovasculoductal injuries. Kim et al. [44] further highlight the importance of ultrasound in preventing complications, such as parotid gland and duct injuries, during thread lifting. They demonstrate how Doppler ultrasound can be used both preoperatively and intraoperatively to map out the precise locations of the parotid gland and other critical structures, allowing for safer thread insertion. The study also shows that ultrasound can detect complications, such as gland perforation, promptly, enabling immediate corrective actions. These findings underscore the importance of integrating ultrasound imaging into cosmetic procedures to enhance accuracy, reduce the risk of adverse outcomes, and ensure patient safety.

Lastly, thread lifting involves the engagement of facial retaining ligaments. These structures are pivotal as they provide the necessary support for the placement and tension of the threads. Knowing how to utilize these ligaments effectively is essential for achieving a durable and effective lift without over‐tightening the skin or causing unnatural distortions. The correct engagement of these ligaments can make a significant difference in the longevity and quality of the lifting effect, offering patients results that are not only immediately noticeable but also lasting.

In conclusion, thread lifting is a complex aesthetic procedure that integrates advanced knowledge of facial anatomy with technical skill. For clinicians, continuous education and practical experience in these anatomical aspects are indispensable for ensuring that thread lifting not only meets aesthetic goals but also preserves the natural function and safety of the facial structures involved. As the technique evolves, so does the need for refined skills and deeper anatomical understanding, making thread lifting a dynamic and highly specialized field in cosmetic medicine.

## Author Contributions

All authors have reviewed and approved the article for submission. Conceptualization: Gi‐Woong Hong, Kyu‐Ho Yi, Soo Yeon Park, and Lisa Kwin Wah Chan. Writing – original draft preparation: Gi‐Woong Hong, Kyu‐Ho Yi, Soo Yeon Park, Jovian Wan, and Kar Wai Alvin Lee. Writing – review and editing: Gi‐Woong Hong, Kyu‐Ho Yi, Soo Yeon Park, Jovian Wan, and Kar Wai Alvin Lee. Visualization: Gi‐Woong Hong, Kyu‐Ho Yi, Soo‐Bin Kim, Youngjin Park, and Olena Sydorchuk. Supervision: Gi‐Woong Hong and Kyu‐Ho Yi.

## Disclosure

The authors have nothing to report.

## Conflicts of Interest

I acknowledge that I have considered the conflict of interest statement included in the “Author Guidelines.” I hereby certify that, to the best of my knowledge, that no aspect of my current personal or professional situation might reasonably be expected to significantly affect my views on the subject I am presenting.

## Data Availability

The data that support the findings of this study are available from the corresponding author upon reasonable request.
